# Supplementary material for: Cryo-EM structures of human ZnT8 in both outward- and inward-facing conformations
Source: eLife. 2020 Jul 29;9:e58823. doi: 10.7554/eLife.58823 (PMC7428307; doi:10.7554/eLife.58823)
Supplement: Supplementary file 1. [file elife-58823-supp1.doc]

**CryoEM data collection and model statistics.**

|  | HsZnT8-DM  in the absence of Zn  (EMDB-22285)  (PDB 6XPD) | HsZnT8-WT  in the presence of Zn  (EMDB-22286)  (PDB 6XPE) | HsZnT8-WT  in the absence of Zn  (EMDB-22287)  (PDB 6XPF) |
| --- | --- | --- | --- |
| **Data collection and processing** |  |  |  |
| Magnification | 60,241 | 60,241 | 60,241 |
| Voltage (kV) | 300 | 300 | 300 |
| Electron exposure (e^–^/Å^2^) | 60 | 60 | 60 |
| Defocus range (μm) | 0.4-0.6 | 0.4-0.6 | 0.4-0.6 |
| Pixel size (Å) | .83 | .83 | .83 |
| Symmetry imposed | C2 | C2 | C1 |
| Initial particle images (no.) | 1,287,890 | 1,744,656 | 1,537,280 |
| Final particle images (no.) | 55,851 | 419,917 | 74,612 |
| Map resolution (Å)  FSC threshold | 3.8  0.143 | 4.1  0.143 | 5.9  0.143 |
|  |  |  |  |
| **Refinement** |  |  |  |
| Model composition  Non-hydrogen atoms  Protein residues  Ligands | 4622  606  4 | 4292  554  8 | 2308  576  4 |
| R.m.s. deviations  Bond lengths (Å)  Bond angles (°) | 0.007  0.764 | 0.007  1.082 | 0.002  0.655 |
| Validation  MolProbity score  Clashscore  Poor rotamers (%) | 2.18  13.68  0.2 | 2.64  20.00  2.08 | 1.75  5.53  0.00 |
| Ramachandran plot  Favored (%)  Allowed (%)  Disallowed (%) | 90.57  9.43  0.00 | 88.11  10.57  1.32 | 92.83  7.17  0.00 |
